# Some Hematological and Physiological Indicators of Health in Triploid Tambaqui (*Colossoma macropomum*): A Preliminary Study

**DOI:** 10.3390/ani16050797

**Published:** 2026-03-04

**Authors:** Aldessandro da C. Amaral, Lucas S. Torati, Luciana N. Ganeco-Kirschnik, Jéssica A. M. Cruz, Janaína S. I. Valandro, Wallice L. P. Duncan, Velmurugu Puvanendran, Fernanda L. Almeida O’Sullivan

**Affiliations:** 1 Programa de Pós-graduação em Ciência Animal e Recursos Pesqueiros, Universidade Federal do Amazonas (UFAM), Avenida Rodrigo Octávio, Manaus 69080-900, AM, Brazil; aldessandro_costa@hotmail.com; 2 Embrapa Pesca e Aquicultura, Av. NS 10, Cruzamento com a Av. LO 18 Sentido Norte Loteamento-Água Fria, Palmas 77008-900, TO, Brazil; lucas.torati@embrapa.br (L.S.T.); luciana.ganeco@embrapa.br (L.N.G.-K.); fernanda.almeida@embrapa.br (F.L.A.O.); 3 Instituto Tocantinense de Educação Superior e Pesquisa (ITOP), ACSU SE 40, Lote 16, AV. NS 02, Palmas 77021-634, TO, Brazil; jessicacruzghj@gmail.com; 4 Programa de Pós-graduação em Aquicultura, Aquaculture Center of UNESP-Caunesp, Street Professor Paulo Donato Castellane, s/n, Jaboticabal 14844-900, SP, Brazil; janaina.valandro@unesp.br; 5 Laboratório de Morfologia Funcional, Departamento de Morfologia, Universidade Federal do Amazonas, Manaus 69067-005, AM, Brazil; wduncan@ufam.edu.br; 6 Center for Marine Aquaculture, Nofima AS, Muninbakken 9, 9019 Tromsø, Norway

**Keywords:** triploidy, *Colossoma macropomum*, hematology, aquaculture, erythrocyte

## Abstract

This study evaluated the physiological condition and health status of triploid tambaqui (*Colossoma macropomum*) using hematological and biochemical indicators. Hematocrit, hemoglobin concentration, erythrocyte count, glucose, and plasma protein levels (albumin, globulin and albumin/globulin ratio) were assessed in triploid and diploid fish, and mean corpuscular volume (MCV), mean corpuscular hemoglobin (MCH), and mean corpuscular hemoglobin concentration (MCHC) were calculated. Triploid fish exhibited larger erythrocytes, reduced erythrocyte counts, and slightly lower hemoglobin and hematocrit values compared with diploids. Correspondingly, MCV and MCH were significantly higher in triploids, indicating increased hemoglobin content per erythrocyte. In contrast, MCHC values did not differ between ploidy groups, suggesting that hemoglobin concentration within erythrocytes remained unaffected. No significant differences were observed in the evaluated biochemical parameters. These findings indicate that triploid tambaqui undergo compensatory physiological adjustments associated with increased nuclear DNA content in erythrocytes, enabling the maintenance of efficient oxygen transport and respiratory capacity. Overall, triploid fish displayed normal physiological performance, comparable to that of diploids. The results support the use of triploidy as a viable and sustainable strategy in tambaqui aquaculture.

## Simple Summary

This study provides the first comprehensive hematological and biochemical evaluation of triploid tambaqui, demonstrating that triploidy does not compromise physiological health or metabolic stability. By showing that triploid fish exhibit compensatory adjustments in erythrocyte structure that preserve oxygen transport and overall respiratory capacity, the work establishes a physiological basis for the safe and sustainable application of triploidy in tambaqui aquaculture. These findings support the use of triploidization as both a production-enhancing and biosafety strategy, contributing valuable evidence for its broader adoption in tropical aquaculture systems.

## 1. Introduction

With the stagnation of capture fisheries since the 1990s, coupled with population growth, aquaculture emerged as an alternative to meet the demand for fish protein. Thus, global production of farmed fish has been steadily increasing and intensifying. However, to achieve satisfactory results, several factors must be carefully considered and balanced in fish farming, such as the selection of the species to be farmed, which is particularly critical. Tambaqui (*Colossoma macropomum*), a characid native to the Amazon basin, presents key characteristics that support its cultivation potential, including rapid growth, physiological and anatomical adaptation to environments with low oxygen concentrations [1,2], tolerance to high densities [3,4,5], and a large market, mainly in the North region of Brazil. Despite these favorable traits, tambaqui farming systems and technological development remain significantly behind those established for other fish species that dominate Brazilian aquaculture and serve as a benchmark in both national and international contexts, such as Nile tilapia (*Oreochromis niloticus*) [6]. This disparity underscores the need to intensify research efforts aimed at advancing and innovating technologies for native species such as tambaqui, with a focus on strategies that confer competitive advantages to the sector.

Considering techniques to increase productivity, the farming of triploid fish is widely practiced in countries where aquaculture is already a consolidated industry, such as Australia (salmon *Salmo salar*) [7] and Scotland (trout *Oncorhynchus mykiss*) [8]. In other countries, some fish species have also demonstrated 100% efficiency in obtaining triploids, among them the channel catfish *Ictalurus punctatus* (USA) [9], coho salmon *Oncorhynchus kisutch* (Croatia and Norway) [10,11], European catfish *Silurus glanis* (Czech Republic) [12], the neotropicals jundiá *Rhamdia quelen* (Brazil) [13,14] and matrinxã *Brycon amazonicus* (Brazil) [15]. The production of triploid fish is very advantageous in the farming process, generating economic profits, mainly because they are sterile individuals. For tambaqui, there were some attempts to induce triploidy through temperature shock with 17 to 59% induction [16,17,18] and with hyperbaric pressure with 100% induction [19]. These positive factors of triploidy, added to the advantageous economic characteristics of tambaqui, can add more value to the production of the species, allowing a high yield in a shorter period.

Hematological examination is one of the commonly used methods to assess the physiological status and health of fish [20,21]. Hematological and biochemical indices provide comprehensive information about the oxygen transport capacity of fish, immunological potential, stress level, disease, intoxication and nutritional status [22].

While enhanced growth is often cited as a potential benefit of triploidy, it is neither universal nor required for its aquaculture relevance. One of the principal advantages of triploidy is functional sterility, which is increasingly valued in aquaculture, not only for its possible effects on energy allocation and production efficiency, but also for its critical role in genetic containment. Sterile stocks are particularly important for protecting elite selective-breeding lines and emerging biotechnological products, including gene-edited or transgenic fish, and for reducing ecological risks associated with escapees.

In this context, the demonstration that triploid tambaqui maintain normal physiological homeostasis provides essential baseline evidence supporting their feasibility for commercial and environmental management applications, even in the absence of immediate growth advantages.

As part of the goal of developing triploidy technology for tambaqui, the present study was aimed at assessing the physiological condition and health status of triploid individuals through comprehensive hematological analyses, hence ensuring that the triploidy condition does not compromise the overall health and welfare of the fish.

## 2. Materials and Methods

### 2.1. Artificial Reproduction and Experimental Design

The study was conducted at the Embrapa Pesca e Aquicultura (Palmas-TO, Brazil). On 8–9 November 2023, 3.5-year-old male (*n* = 3, 4.5 ± 0.5 kg) and female (*n* = 2, 8 kg) tambaqui were selected and treated with carp pituitary extract (CPE, Danube Piscicultura, Garcia, Blumenau, SC, Brazil) [23], to induce ovulation, spawning and spermiation. Females received a preparatory intraperitoneal dose of 0.5 mg kg^−1^ body weight (BW) and, 12 h later, a dose of 5.0 mg kg^−1^ BW, and males were treated with a single intraperitoneal dose of 2.5 mg kg^−1^ BW. When females were releasing eggs at 260 degree-hours, they were anesthetized and stripped for the release of the eggs [24]. The color of the eggs was checked, which was homogeneously light green and free of any white spots (dead/overripe eggs). At the same time, males had their urinary tract emptied, the urogenital papillae dried, and semen was collected using sterile 5 mL dry syringes. Sperm was examined under an optical microscope to confirm whether any urine/feces contamination resulted in sperm activation, which would lead to reduced, or no, fertilization [25]. Equal amounts of semen from three males (2 mL/male) were then pooled and gently mixed prior to fertilization. Likewise, equal amounts of eggs (200 g/female) from the two females were pooled prior to fertilization.

The treatment was performed in triplicate, and each replicate consisted of 12.5 g of pooled eggs fertilized with 250 µL of pooled semen inside a 50 mL Falcon tube with perforated caps to prevent pressure build-up inside the Falcon tube during the pressure treatment. The Falcon tubes containing fertilized eggs were placed together in the stainless-steel chamber of the hydrostatic pressure shock machine (TRC Hydraulics, Dieppe, NB, Canada), which was filled with incubator water (29 °C). Hence, 95 s after fertilization (SPF) the eggs were submitted to a pressure of 8000 psi for 90 s [19]. Immediately after the pressure treatment, decompression was performed instantly, using a pressure relief valve. Control (C) replicates were fertilized in Falcon tubes similar to the treatment, but were not subjected to any pressure. All fertilized eggs in each of the Falcon tubes were incubated in individual 200 L incubators (*n* = 6).

### 2.2. Fertilization Index (FI) and Embryo Survival (Hatching) Index (ESI)

To estimate the fertilization index (FI) and embryo survival index (ESI), a random triplicate samples of 5 mL water sample containing embryos (at final gastrula stage) were taken from each incubator at 6 h post fertilization (hpf) for FI estimation and 11 hpf for ESI estimation, and were placed in a Petri dish to form a single layer of embryos. To estimate FI, the number of fertilized and unfertilized eggs were counted under a binocular microscope and FI was calculated as FI = (number of fertilized eggs/total number of eggs in the sample) × 100. To estimate the ESI, the number of moving embryo inside the egg (viable) and whitish eggs or with dead embryos (nonviable) were counted, and the ESI was calculated as ESI = (number of viable eggs/total number of eggs) × 100. The stage of viable embryos at 11 hpf is considered as a good proxy for the number of hatched larvae [26]. Larval hatching started approximately 12 h after fertilization, at 28 °C (336 degree-hours).

### 2.3. Rearing Conditions and Feeding Regime

The larvae were kept in the incubators and from the 5th day post hatching (dph) they were fed with vitellus of brine shrimp eggs (Vitellus; BernAqua HatcheryFeeds; Olen, Belgium) every two hours. From 15 to 20 dph, the juveniles were co-fed ad libitum with commercial powdered feed (50% crude protein, Aqua line SUPRA, São Leopoldo, RS, Brazil), for weaning. Subsequently, a total of 1956 treated larvae and 2002 control larvae were transferred to 15,000 L outdoor vinyl tanks, previously fertilized with rice bran (750 g) and simple superphosphate (500 g). They were kept in a partial water renewal system under constant aeration. Water quality was maintained at ideal conditions (Oxigen 6 mg L^−1^; pH—7.5) for the species [27]. The average temperature was 29 °C, and 12 h of light per day (12L:12D). Larvae were completely weaned onto a dry diet at 20 dph. From that point until 16 months of age, all fish were fed a commercial diet containing 45% crude protein (Aqua line SUPRA, São Leopoldo, Brazil), with feed particle size adjusted according to the developmental stage, offered daily at 10% of the estimated total biomass.

### 2.4. Blood Smear

To identify and confirm triploid individuals, the longest diameter of erythrocytes from 12-month-old fish was measured. For this, 70 control fish and 70 treated fish received individual electronic pit tags (AnimallTag, São Carlos, SP, Brazil) and were sedated with 100 mg L^−1^ tea tree essential oil (*Melaleuca alternifolia*) (Ferquima, Vargem Grande Paulista, SP, Brazil) for blood sampling, to verify the ploidy status. Whole blood, collected in heparinized syringes, was immediately used to prepare blood smears, which were air-dried and stained with Giemsa. Images of erythrocytes were obtained under an optical microscope at 100× magnification, and the diameter of 30 erythrocytes per sample was measured using Image J software (v. 1.8) to identify triploid fish [19].

### 2.5. Sampling

At 16 months of age, 30 control fish and 35 triploid fish were sampled for biometric and hematological analyses. Biometric data were recorded for body weight (W, g), total length (TL, cm), and standard length (SL, cm). Fish were deeply sedated with 100 mg L^−1^ of tea tree (*Melaleuca alternifolia*) essential oil, and blood was collected from the caudal vein with a syringe rinsed with 10% EDTA.

### 2.6. Biochemical Parameters

Immediately after collection, the blood samples were centrifuged at 2500 rpm for five minutes. Plasma was then collected and stored at −80 °C until analysis, following standard procedures commonly applied in fish physiology [28]. The biochemical variables analyzed were glucose, determined using an enzymatic colorimetric test (InVitro^®^, Belo Horizonte, Brazil); total protein, measured using the biuret method [29]; and albumin, determined using the bromocresol green method (colorimetric determination; InVitro^®^, Belo Horizonte, Brazil) [30]. Serum globulin concentration was estimated by subtracting albumin from total plasma protein, as commonly applied in teleost fish biochemical assessments [31]. The albumin/globulin (A/G) ratio was calculated by dividing albumin concentration by globulin concentration, according to the equation: A/G = albumin/(total proteins − albumin) [31].

### 2.7. Hematological Parameters

Hematocrit and hemoglobin rates were analyzed as described by Goldenfarb et al. (1971) [32] and Drabkin (1948) [33], respectively. Erythrocytes were counted in a Neubauer chamber under a 40× objective of an optical microscope (Leica 2500, São Paulo, Brazil), after blood dilution in citrate formaldehyde (Collier, 1944) [34]. Then, it was possible to estimate mean corpuscular volume [MCV = (hematocrit × 10)/erythrocytes], mean corpuscular hemoglobin [MCH = (hemoglobin × 10)/erythrocytes] and mean corpuscular hemoglobin concentration [MCHC = (hemoglobin rate × hematocrit) × 100], according to Wintrobe (1934) [35].

### 2.8. Statistical Analysis

Fertilization index, embryo survival index, fish length and weight, biochemical (glucose, total protein, albumin, serum globulin and albumin/globulin ratio), hematimetric indices (MCV, MCH and MCHC) and hematological parameters (hematocrit, hemoglobin and erythrocyte number) were checked for normal distribution and homogeneity of variance using the Shapiro–Wilk and Levene tests, respectively. The equality of the remaining data was analyzed using the *t*-test to compare the treatment mean with that of the control in all analyses (confidence limit of *p* < 0.05). Data are represented as mean ± standard deviation (SD). Statistical analyses and graphical outputs were performed using RStudio (version 2025.05.0+496; R 4.5.0 for Windows). The RVAideMemoire, car, and dplyr packages were used for data manipulation and analytical procedures.

## 3. Results

### 3.1. Fertilization Index (FI) and Embryo Survival (Hatching; ESI) Indices

**Figure 1:**
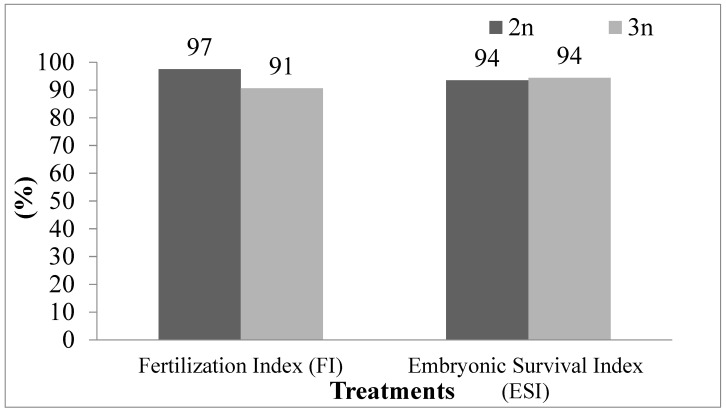
Fertilization index (FI) and embryonic survival index (ESI) in tambaqui (*Colossoma macropomum*) with different ploidies: diploid (2n, control) and triploid (3n, triploid).

The fertilization rate was 97% in the control group and 91% in the triploid group, with a significant difference between the means (t (4) = 3.5496; *p* = 0.02381). The embryonic survival rate for both groups was 94%, with no significant difference between the means (t (4) = 0.3015; *p* = 0.778) (Figure 1).

### 3.2. Blood Smear

**Figure 2:**
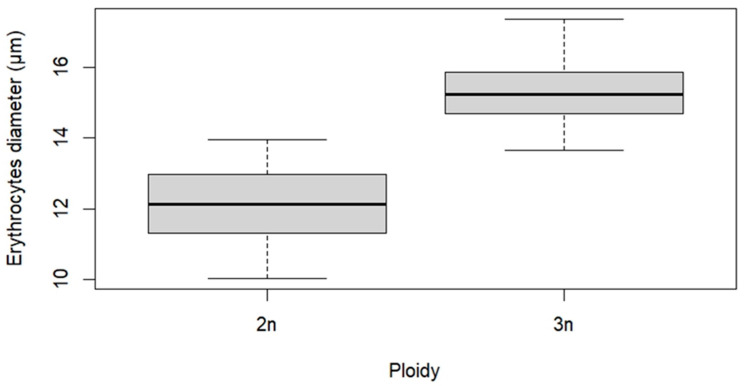
Erythrocyte size of tambaqui (*Colossoma macropomum*) with different ploidy levels. Triploid individuals showed higher median values and a distribution shifted toward larger cell diameters compared to diploids, indicating increased erythrocyte size associated with ploidy level.

The erythrocytes of diploid fish exhibited an average length of 12.1 ± 0.9 µm, whereas those of fish induced to triploidy showed a significant (t (123) = −19, *p* = < 2.2e^−16^) larger average size of 15.3 ± 0.8 µm, consistent with the expected increase in cell dimensions associated with triploidy (Figure 2).

### 3.3. Biometrics

**Figure 3:**
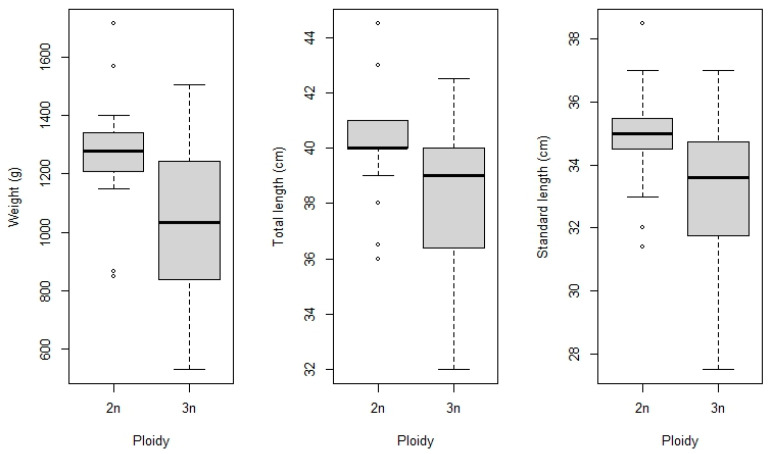
Weight, total length, and standard length of tambaqui (*Colossoma macropomum*) with different ploidy levels. Diploid individuals tended to show higher median values for all morphometric variables, while triploids exhibited greater within-group variability. Points represent outliers.

At 16 months of age, the fish were sampled for biometrics (Figure 3). The mean weight of the control group was 1268 g ± 203.2, and that of the triploid group was 1042 g ± 269.9. The *t*-test showed a significant difference between the means of the groups (t (43) = 3.192; *p* = 0.00267). For total length, the mean of the control group was 40.06 cm ± 1.98 and that of the triploid group was 38.00 cm ± 2.72. The *t*-test showed no significant difference between the means (t (43) = 2.5074; *p* = 0.01602). For the standard length, the mean for the control was 34.88 cm ± 1.98, and for the treated group it was 33 cm ± 2.42, with no significant difference between the means (t (43) = 2.4953; *p* = 0.0165).

### 3.4. Biochemical Parameters

**Figure 4:**
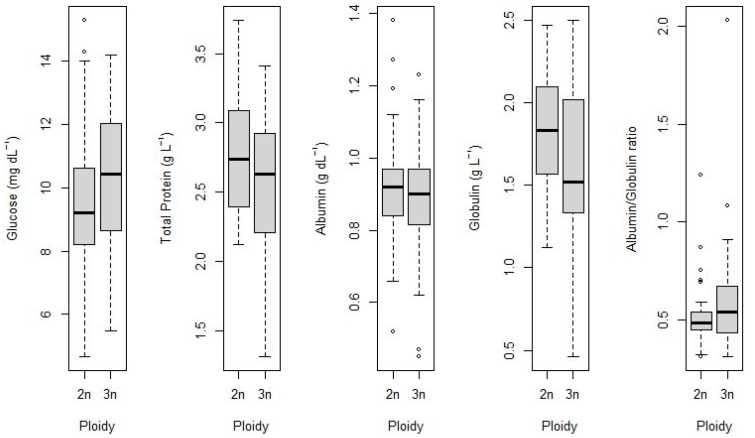
Biochemical parameters of tambaqui (*Colossoma macropomum*) with different ploidy levels. In general, diploid individuals showed slightly higher medians for most biochemical parameters, except for glucose and albumin/globulin ratio, while triploid individuals showed greater intragroup variation in some variables. Points represent outliers.

Blood plasma biochemical analyses indicated no statistically significant differences between triploid tambaqui (*n* = 35) and diploid controls (*n* = 30), as reflected by comparable mean levels of glucose, total protein, albumin, serum globulin and albumin/globulin ratio (*t*-test; *p* > 0.05; Figure 4). The glucose mean was 10.36 ± 2.40 mmol/L in the triploid group and 9.64 ± 2.48 mmol/L in the control group, with no significant difference between the means (t (63) = 1.20, *p* = 0.23). The mean total protein of the triploid group was 2.53 ± 0.52 g/dL, and of the control group it was 2.75 ± 0.41 g/dL, with no significant difference between the means (t (63) = 1.88, *p* = 0.06). The albumin mean values were 0.89 ± 0.17 and 0.92 ± 0.17 g/dL for the triploid and control groups, respectively (t (63) = 0.83, *p* = 0.40). The globulin was 1.64 ± 0.47 and 1.83 ± 0.37 g/dL for the triploid and control groups, respectively (t (63) = 1.78, *p* = 0.07).

### 3.5. Hematological Parameters

**Figure 5:**
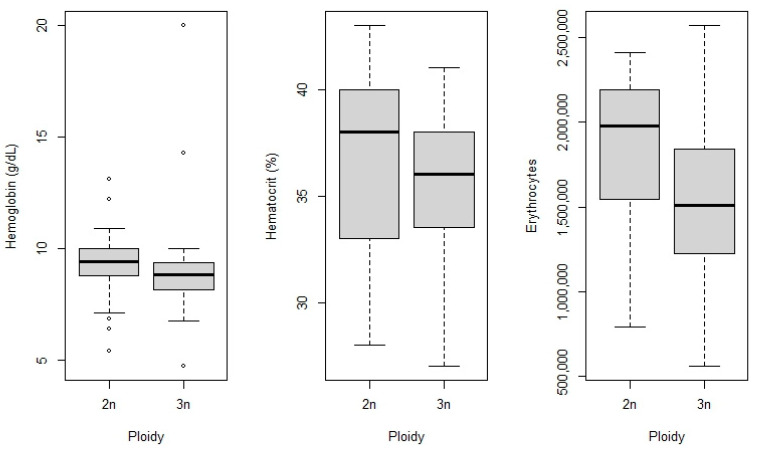
Hematological parameters of tambaqui (*Colossoma macropomum*) with different ploidy levels. Diploid individuals tended to present higher median values for all hematological parameters, whereas triploids showed wider variability in some measurements. Points represent outliers.

Blood samples were collected from 65 fish: 30 from the control group (2n) and 35 from the triploid group (3n). The mean hematocrit of triploid fish (35.82 ± 3.24%) was similar to the mean of the control group (37 ± 3.95%) (t (51) = 1.17; *p* = 0.24). The hemoglobin means were also similar between triploid (9.18 ± 2.69 g dL^−1^) and control fish (9.45 ± 1.88 g dL^−1^) (t (42) = 0.25; *p* = 0.79). The average number of erythrocytes per µL was lower in triploid (1.5 ± 3.24 × 10^6^) than in control (1.8 ± 3.95 × 10^6^) (t (53) = 2.82; *p* = 0.006 (Figure 5).

**Figure 6:**
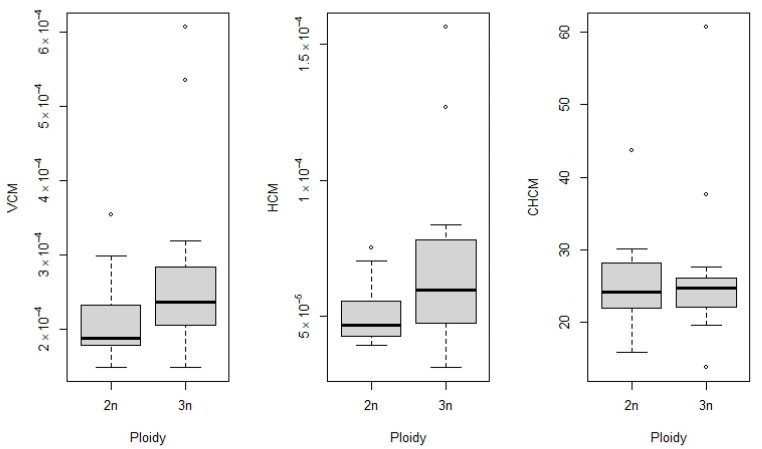
Hematimetric indices of tambaqui (*Colossoma macropomum*) with different ploidy levels. Comparison of VCM, HCM, and CHCM between diploid (2n) and triploid (3n) fish. Triploids showed higher values of mean corpuscular volume (VCM) and mean corpuscular hemoglobin (HCM), whereas mean corpuscular hemoglobin concentration (CHCM) remained relatively similar between ploidy groups. Points represent outliers.

Regarding the hematimetric indices, the triploid group presented a significantly higher mean corpuscular volume—MCV (0.00025 ± 0.0001) than the control group (0.00021 ± 0.00005) (t (36) = −2.42, *p* = 0.020), a significantly higher mean corpuscular hemoglobin—MCH (0.000063 ± 0.000025) than the control group (0.000051 ± 0.00002) (t (33) = 2.48, *p* = 0.017), and a nonsignificant mean corpuscular hemoglobin concentration—MCHC 25.61 ± 8.06 (3n) and 25.25 ± 5.04 (2n, (t (43) = 0.19, *p* = 0.84) (Figure 6).

## 4. Discussion

Hematology provides simple, yet reliable, methods for assessing the physiological status of fish, offering valuable insights into various biological processes. It serves as a good indicator of fish welfare and health, and, indirectly, of the environmental conditions to which they are exposed. This study aimed to evaluate the physiological condition and health status of triploid tambaqui through detailed hematological analyses. These assessments were conducted to ensure that the triploid condition does not adversely affect the fish’s overall health or welfare, thereby supporting the sustainable application of triploidy as a biotechnological tool to enhance tambaqui production.

Measuring erythrocytes through blood smears is a rapid and inexpensive method widely used to determine ploidy in teleosts [36,37]. However, additional analyses, such as flow cytometry and cytogenetics, are often required to validate the results and confirm triploidy. In a previous study, we confirmed the correlation between erythrocyte size, flow cytometry and karyotype in diploid and triploid tambaqui, demonstrating that erythrocyte diameter measurement is a validated and reliable method for identifying triploid tambaqui [19]. Hence, in this study, we used the erythrocyte diameter, measured in blood smears, to confirm the triploid tambaqui.

Our results show that the number of erythrocytes in triploid tambaqui was lower than in diploids. This reduction results from an increase in erythrocyte size and, consequently, the decrease in their number, as also reported in other species [38,39]. The extra genetic material leads to reduced erythrocyte counts in triploid organisms, a homeostatic mechanism that compensates for the increase in cell volume [36,40,41].

Although the present study was primarily designed to evaluate hematological and biochemical indicators at a standardized production stage (16 months of age), biometric parameters (body weight, total length, and standard length) were recorded and are presented in Section 3.3. No significant differences in total or standard length were observed between diploid and triploid tambaqui at 16 months of age, although diploid individuals exhibited significantly higher mean total weight. This difference may be associated with greater energy expenditure related to respiratory gas transport and metabolic activity in triploid fish [36,42]. Similar patterns have been observed in *Oncorhynchus mykiss* [36], *Cyprinus carpio* [38], and *Clarias gariepinus* [42], where triploid fish showed lower erythrocyte counts and relatively slower growth, possibly due to reduced oxygen transport efficiency and increased energy expenditure. In an earlier study by this research group [19], it was demonstrated that triploidy can influence somatic growth patterns, particularly body weight gain and growth rate during the early stages of development. The article reports that triploid individuals may exhibit differences in weight gain trajectories compared to diploids, even when length parameters remain similar. Since we have reported the advantages of triploid ploidy for the species in the early stages of development in the above-mentioned article, we evaluated physiological homeostasis at a defined production stage. Importantly, the evaluation of hematological and biochemical parameters provides a necessary physiological context for interpreting growth outcomes and assessing the feasibility of triploidy as a management or biotechnological tool. These data establish a critical foundation for future applied studies in tambaqui farming, including optimization of rearing strategies and evaluation of potential advantages of triploid lineages beyond growth performance. However, the collection of repeated intermediate samples (e.g., at 3 or 6 months) was not included in the experimental design to avoid handling-induced stress, which could confound hematological and biochemical parameters.

No significant differences were observed in hemoglobin (Hb) and hematocrit values, although mean values were slightly lower in triploid tambaqui, which may reflect subtle physiological adjustments in oxygen transport efficiency without compromising growth or overall condition. Red blood cells are directly involved in oxygen and carbon dioxide transport through their main component, hemoglobin, while hematocrit represents the percentage of blood volume occupied by these cells [36,43]. Thus, both the number and volume of red blood cells indicate the blood’s oxygen-carrying capacity [43,44]. The Hb content and hematocrit are regarded as key indicators of the secondary stress response.

Regarding hematometric indices, mean corpuscular volume (MCV) and mean corpuscular hemoglobin (MCH) were higher in triploid fish compared to diploids, reflecting higher hemoglobin content per cell, possibly due to the greater DNA content in triploid red blood cells. Mean corpuscular hemoglobin concentration (MCHC), which represents the ratio of hemoglobin quantity to cell volume, showed similar values between diploids and triploids, indicating that this function was not compromised in triploid fish. Hence, although triploid erythrocytes are morphologically larger, the internal hemoglobin concentration remains within a stable physiological range, suggesting compensatory hematological efficiency between the groups [43,44].

Biochemical parameters showed no differences in triploid tambaqui compared to the control group, indicating no effects in plasma glucose levels, total protein, albumin, serum globulin and albumin/globulin ratio caused by triploid condition. This indicates that, in triploid tambaqui, the activation of stress response mechanisms, such as cortisol and catecholamine release, which stimulate gluconeogenesis and the mobilization of energy reserves [36,42] and the immune response [45], remained stable between ploidies, and may not be influenced by chromosomal variation. Altogether, these results indicate that physiological homeostasis may have been preserved in triploid tambaqui.

## 5. Conclusions

The results of this study indicate that triploidy in *Colossoma macropomum* does not induce significant hematological or biochemical alterations that would compromise fish health or physiological performance. Although triploid individuals exhibited larger erythrocytes, reduced erythrocyte counts, and slightly lower hemoglobin and hematocrit values, these changes appear to reflect compensatory physiological adjustments that maintain effective oxygen transport and overall respiratory capacity. Likewise, the stability of glucose, total protein, globulin, and albumin concentrations and albumin/globulin ratio indicate that triploid fish are able to preserve biochemical homeostasis, even under increased metabolic demands or physiological stress. Collectively, these findings demonstrate that triploid tambaqui maintain physiological function and health status comparable to those of diploid individuals, supporting triploidy as a safe and sustainable biotechnological strategy to enhance aquaculture production without compromising animal welfare. Nevertheless, further studies under more physiologically demanding conditions are warranted to better elucidate the limits of these compensatory mechanisms. Importantly, triploidy has not previously been investigated in any neotropical fish species. To our knowledge, this study provides the first integrated hematological and biochemical characterization of triploid *Colossoma macropomum*, a species of major importance for neotropical aquaculture. By establishing baseline physiological profiles and identifying triploidy-associated alterations in this species, our findings extend beyond descriptive confirmation and provide critical functional information necessary to evaluate the feasibility and potential advantages of triploid lineages in tropical aquaculture systems and physiological homeostasis at a defined production stage. We recognize that future studies, combining longitudinal growth curves with physiological profiles, would provide a more comprehensive understanding of the effects of ploidy in *Colossoma macropomum*.

## Data Availability

The original contributions presented in this study are included in the article. Further inquiries can be directed to the corresponding author.
